# Chemotherapeutic Effect on Ovarian Stroma and Tumor Cells Which Make Problem in Diagnosis and Staging of Tumor 

**DOI:** 10.30699/ijp.2021.520513.2540

**Published:** 2021-07-06

**Authors:** Neda Nasirian, Khadigeh Elmizadeh

**Affiliations:** 1 *Department of Pathology, Qazvin University of Medical Sciences, Qazvin, Iran *; 2 *Department of Obstructive, Qazvin University of Medical Sciences, Qazvin, Iran *

**Dear Editor**

We had a case of Krukenberg tumor (KT) of the right ovary, which extended to pelvic zone M;18*13*10 cm in a 40-year-old virgin woman who had no history of previous cancer. The patient received five courses of chemotherapy before surgery, and because of the chemotherapeutic effect on the ovarian stroma, spindle cells of stroma were plump, hyperchromatic, with mild pleomorphism. The number of signet cells was low, and they were scattered between spindle cells, mostly with eosinophilic cytoplasm. Spindle cell tumors such as pelvic rhabdomyosarcoma (with scattered eosinophilic rhabdoid cells) also gastrointestinal stromal tumor (GIST) with signet ring-like change were in the differential diagnosis in frozen section examination and permanent sections. Because of the increasing use of preoperative chemotherapy in the management of ovarian malignancy, pathologists should be aware of changes in cellular morphology induced by chemotherapy, which may cause problems in tumor typing and grading and the identification of ovarian epithelial tumors or metastatic tumors to the ovary such as breast and lung cancers (1). Clinically, the chemotherapy effect on ovary can be different from none to some levels of partial damage and reduced fertility, until the totally ovarian damage and complete loss of primordial follicles, ovarian atrophy or failure (2).

Effects of chemotherapeutic drugs on the cellular stroma of the ovary were seen in the literature previously, which can cause apoptotic depletion of follicles (3). Chemotherapeutic drugs such as cyclophosphamide, cisplatin, and doxorubicin, can cause insufficiency of the ovary in many cases in the form of atresia of growing follicles and death of primordial follicles (4). Also, damage to vessels and stromal cells can be seen (4-5). They also can induce inflammation. Tumor cells morphology can change, including decreased nucleocytoplasmic ratio or enlarged nuclei with very irregular nuclear border or degeneration of nuclei with smudging or clumping of chromatin and intense eosinophilia cytoplasm (6). 

Age of women at menopause show duration of estrogen deficiency which can cause health problems (7). The tumor stroma not only provides nutrients and growth factors for the tumor but also helps with the reprogramming of tumoral cells and acts as a barrier against chemotherapy. Stromal cells of tumors can signal normal epithelial cells to adapt with tumor cells (8-9). Pronounced stromal changes caused by chemotherapy include stromal fibrosis, infiltration of inflammatory cells, fibrinous debris, cholesterol clefts, hemosiderin pigment, and vascular proliferation. Especially within the omentum, there were large areas of fat necrosis with collections of foam cells. Therefore, the presence of pronounced stromal changes may be a good indicator of previous tumor infiltration (6-7). This means that during tumor grading, none of the mitosis and cellular morphology, which are essential factors and have important prognostic implications, are reliable after previous chemotherapy (8-9).

Then, correct interpretation requires exact evaluation of the morphology of tumoral cells and stromal cells, patient history, and confirmation of diagnosis with immunohistochemistry such as anti-cytokeratin and anti-histiocytic antibodies.
